# GFAP as Astrocyte-Derived Extracellular Vesicle Cargo in Acute Ischemic Stroke Patients—A Pilot Study

**DOI:** 10.3390/ijms25115726

**Published:** 2024-05-24

**Authors:** Timea Forró, Doina Ramona Manu, Ovidiu-Lucian Băjenaru, Rodica Bălașa

**Affiliations:** 1 Doctoral School of Medicine and Pharmacy, “George Emil Palade” University of Medicine, Pharmacy, Science and Technology of Targu Mures, 540142 Targu Mures, Romania; forro.btimea@gmail.com; 2 Center for Advanced Medical and Pharmaceutical Research, “George Emil Palade” University of Medicine, Pharmacy, Science and Technology of Targu Mures, 540142 Targu Mures, Romania; 3 Discipline of Geriatrics and Gerontology, “Carol Davila” University of Medicine and Pharmacy, 050474 Bucharest, Romania; ovidiu.bajenaru@umfcd.ro; 4 National Institute of Gerontology and Geriatrics “Ana Aslan”, 11241 Bucharest, Romania; 5 1st Neurology Clinic, County Emergency Clinical Hospital of Targu Mures, 540136 Targu Mures, Romania; rodica.balasa@umfst.ro; 6 Department of Neurology, “George Emil Palade” University of Medicine, Pharmacy, Science and Technology of Targu Mures, 540142 Targu Mures, Romania

**Keywords:** acute ischemic stroke, astrocyte-derived extracellular vesicles, glial fibrillary acidic protein, western blotting

## Abstract

The utility of serum glial fibrillary acidic protein (GFAP) in acute ischemic stroke (AIS) has been extensively studied in recent years. Here, we aimed to assess its potential role as a cargo protein of extracellular vesicles (EVs) secreted by astrocytes (ADEVs) in response to brain ischemia. Plasma samples from eighteen AIS patients at 24 h (D1), 7 days (D7), and one month (M1) post-symptoms onset, and nine age, sex, and cardiovascular risk factor-matched healthy controls were obtained to isolate EVs using the Exoquick ULTRA EV kit. Subsets of presumed ADEVs were identified further by the expression of the glutamate aspartate transporter (GLAST) as a specific marker of astrocytes with the Basic Exo-Flow Capture kit. Western blotting has tested the presence of GFAP in ADEV cargo. Post-stroke ADEV GFAP levels were elevated at D1 and D7 but not M1 compared to controls (*p* = 0.007, *p* = 0.019, and *p* = 0.344, respectively). Significant differences were highlighted in ADEV GFAP content at the three time points studied (n = 12, *p* = 0.027) and between D1 and M1 (z = 2.65, *p* = 0.023). A positive correlation was observed between the modified Rankin Scale (mRS) at D7 and ADEV GFAP at D1 (r = 0.58, *p* = 0.010) and D7 (r = 0.57, *p* = 0.013), respectively. ADEV GFAP may dynamically reflect changes during the first month post-ischemia. Profiling ADEVs from peripheral blood could provide a new way to assess the central nervous system pathology.

## 1. Introduction

Stroke is the third leading cause of death and disability worldwide [1,2] with high variability as regards etiology, clinical presentation, infarct size, and localization [3]. Ischemic stroke (IS) is the most common, representing 75–80% of all strokes caused by the occlusion of a cerebral artery due to a blood clot [4]. After stroke, a permanent imbalance between the brain’s metabolic needs and the vascular system’s ability to maintain adequate glucose delivery and gas exchange triggers a multicellular response of neuroinflammation, necrosis, and apoptosis of both neurons and glial cells [5].

Glial cells are the primary components of the peri-infarct area. Astrocytes (also referred to as astroglia) are the most abundant glial cell type in the adult central nervous system (CNS) with critical roles in brain homeostasis regulation [6]. Together with other highly specialized and interconnected cells as neurons, microglia, oligodendrocytes, pericytes, and endothelial cells, they form the neurovascular unit (NVU) [7]. After an ischemic insult, the NVU induces the release of various potential molecules into the blood vessels, cerebrospinal fluid (CSF), and extracellular space, resulting in a specific profile of the biomarkers’ content in these body fluids [8]. Although astrocytes are more resilient than other cell types within the NVU to injury [9], their vulnerability increases in response to ischemia, and their functions may be affected throughout all the different post-stroke phases, aiding or hindering recovery [10]. Under these conditions, astrocytes undergo a characteristic change in appearance from the normally bushy form to hypertrophy of their cellular processes, a phenomenon called reactive astrogliosis (also referred to as astrocyte activation), marked by the upregulated glial fibrillary acidic protein (GFAP) in days 1–4 post-ischemia [11,12]. Reactive gliosis is a gradual reaction, leading to a mature glial scar formation until the chronic phase days 8–14 post-ischemia [11].

The GFAP is the main intermediate filament protein of the astroglia cytoskeleton, which has been classically used as a marker of astrocytes, cellular integrity, and reactive gliosis [13,14,15]. Since its discovery 50 years ago, multiple alternative GFAP gene splice variants have been described, leading to the expression of different—twelve human and seven murine—GFAP isoforms [16]. The GFAP is highly brain-specific; no relevant extracerebral sources of this protein have been identified, and it is not released under physiological conditions. Therefore, blood GFAP levels of healthy individuals are very low, with concentrations below the lower detection limit [17]. GFAP release into the bloodstream reflects glial stress or injury, and elevated serum levels of this protein indicate brain structural damage through cellular destruction [18]. As the GFAP is highly vulnerable to proteolysis, it is released both as an intact protein (approximately 50 kilodaltons, kDa) and as breakdown products (BDPs: 18–44 kDa) derived from caspase- and calpain-cleavage into the blood [19]. Despite some limitations, GFAP has been suggested to be related to traumatic brain injury (TBI) [20,21] and glioblastoma multiforme [22,23,24], and it is a feasible strategy to identify patients with intracranial hemorrhage in the hyperacute phase of stroke [25,26,27,28,29,30,31,32,33]. In IS, the utility of GFAP has also been extensively studied in recent years [34]. Data have shown that serum GFAP levels increase with neurological deficit and lesion size, reflect the severity of the stroke, and improve the ability of the National Institutes of Health Stroke Scale (NIHSS) score to predict poor functional outcomes [35]. Early plasma GFAP can predict clinical and neuroimaging outcomes of IS after successful recanalization [36].

Emerging data suggest that extracellular vesicles (EVs) can be useful diagnostic, prognostic, and therapeutic markers in stroke [37]. These vesicles represent a broad family of cell-derived, lipid-bilayer membrane-enclosed particles secreted by all types of cells into extracellular fluids that can freely pass the blood–brain barrier (BBB) and contain unique markers and content linked to their cell of origin. They play essential roles in cell-to-cell communication, delivering proteins, lipids, and nucleic acids to target cells [38,39,40]. These unique qualities make them novel candidates for non-invasive blood-based biomarkers of CNS cell-specific changes during stroke progression and recovery [41]. Exosomes (30–150 nm) are a subtype of EVs together with microvesicles (ectosomes, 10–1000 nm) and apoptotic bodies (50–5000 nm), which are differentiated based on their biogenesis, release pathways, size, content, and function [42].

Research in the field of EVs has primarily focused on stem cell-derived EVs. Recently, cell-type-specific EVs have garnered attention as they permit highly targeted cell-specific communication due to their molecular composition and unique biogenesis [43]. While studies profiling the cargo of different brain-derived EVs are continuously emerging, a vast minority link EV content to specific cell types within the CNS. Furthermore, literature on post-stroke EV cargo is predominantly focused on microRNAs, and investigations of the protein content are relatively rare [41]. Recent evidence suggested that astrocyte-derived EVs (ADEVs) mediate numerous biological processes in stroke, including neuroprotection and neurorepair [44]. In this pilot study, we aimed to release an ADEV profile of IS patients, focusing on the GFAP as the cargo protein of these EVs in dynamics: 24 h (D1), 7 days (D7), and one month (M1) following the onset of IS. While plenty of studies characterize serum GFAP levels as a potential biomarker in predicting stroke severity and outcome, to the best of our knowledge, this is one of the first papers characterizing ADEVs’ protein cargo in stroke patients.

## 2. Results

### 2.1. Baseline Features of the Study Population

The current study prospectively analyzed eighteen acute ischemic stroke (AIS) patients enrolled within 24 h from symptom onset. The mean age at study inclusion was 66 ± 7.5 years (ranging from 51 to 78). There were eight females, and the median clinical stroke severity was 7.5 (ranging from 6 to 11) on the NIHSS. Five of the nine thrombolysed patients had an ischemic lesion on the craniocerebral computed tomography (CT) scan performed 24 h after stroke onset. Over 80% of the patients presented moderate to severe disability based on the modified Rankin Scale (mRS) at D7 that improved until M1. Nine age, sex, and cardiovascular risk factor-matched healthy subjects were also recruited. The mean age in this group was 65 ± 7 years (ranging from 53 to 74); there were four females. The clinical characteristics of the study population are displayed in Table 1. During the follow-up period, three patients were transferred to other neurorehabilitation facilities and did not manage to come to visit; one had reinfarction, and two others were infected with SARS-CoV-2 one week after being discharged from the hospital. We did not exclude these patients from this study, but in these cases, we did not collect blood samples one month after the stroke onset.

### 2.2. Identification of the Plasma EVs via Bead–Antibody–EVs–FITC Complexes and Flow Cytometry Analysis of Beads-Captured EVs

**Figure 1:**
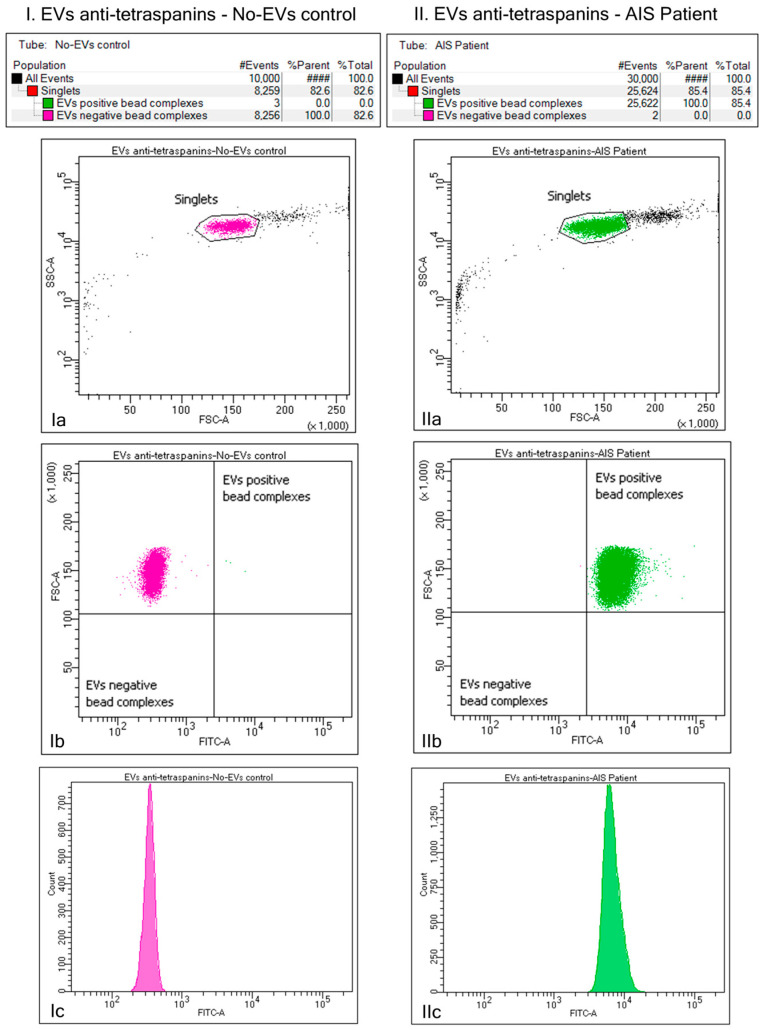
Bead flow separation data for the tetraspanin captured antibodies coupled with Exo-FITC staining. The first column (**I**) depicts beads with no captured extracellular vesicles (EVs), while the second column (**II**) depicts beads with captured EVs. Only singlets were considered for analyses (**Ia**,**IIa**). Plots of forward scatter (FSC) versus (vs.) fluorescein isothiocyanate (FITC) intensity showed that 100% of the particles were FITC-negative in the no-EVs control (**Ib**). In comparison, 100% of the particles were FITC-positive in the EVs-containing sample (**IIb**). A histogram of the fluorescence distribution is also shown for both types of beads (**Ic**,**IIc**).

To confirm the success of the EV isolation process, we assessed the expression of conventional surface EV markers, the tetraspanins (CD9, CD63, CD81), on EVs via flow cytometry (Figure 1). The initially obtained EV suspension was captured using biotinylated antibodies targeting the tetraspanin proteins (anti-CD9, anti-CD63, and anti-CD81) on EV surfaces coupled to Exo-Flow beads. Only singlets were considered during analyses. Plots of forward scatter (FSC) versus (vs.) fluorescein isothiocyanate (FITC) intensity showed that a negligible number of particles were FITC-positive in the no-EVs control, while there were 100% FITC-positive particles in the EVs-containing sample. The analysis showed a pure and rich tetraspanin-positive EV suspension, suggesting that our method was suitable for the purification of EVs.

### 2.3. Purification of ADEV Subpopulation via Bead–Antibody–EVs–FITC Complexes and Flow Cytometry Analysis of Beads-Captured EVs

**Figure 2:**
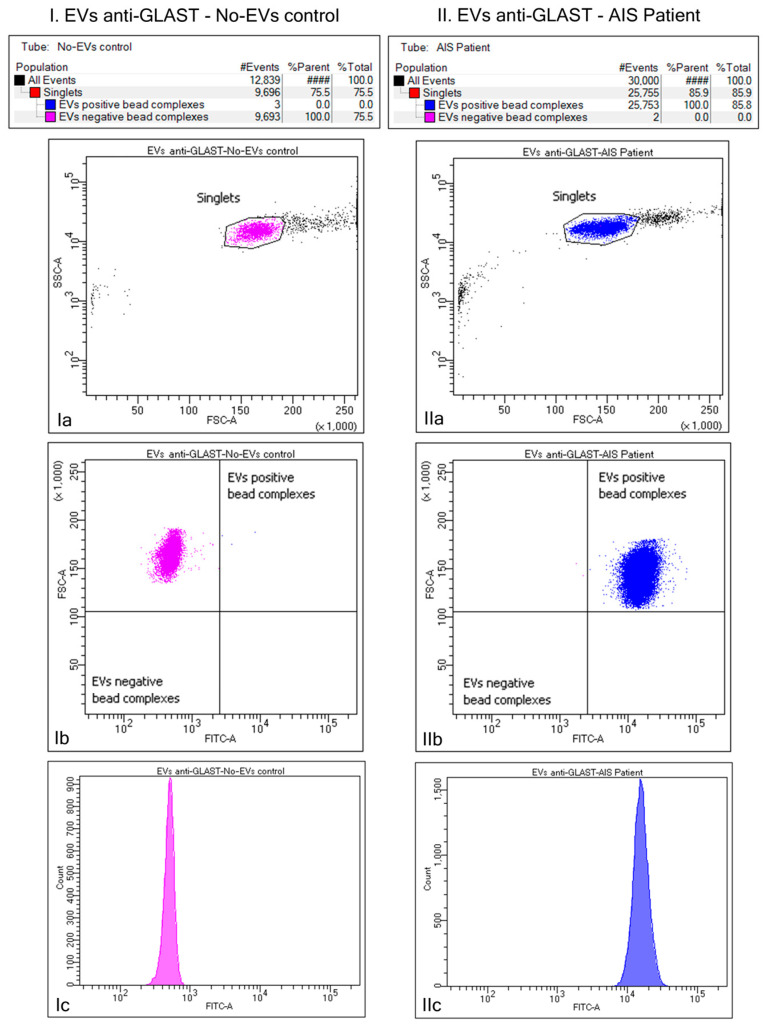
Bead flow separation data for the glutamate aspartate transporter (GLAST) captured antibodies coupled with Exo-FITC staining. The first column (**I**) presents beads with no captured EVs, while the second column (**II**) presents beads with captured EVs. Only singlets were considered for analyses (**Ia**,**IIa**). FSC vs. FITC intensity plots showed that 100% of the particles were FITC-negative in the no-EVs control (**Ib**), while 100% were FITC-positive in the EVs-containing sample (**IIb**). A histogram of the fluorescence distribution is also shown for both beads-types (**Ic**,**IIc**).

To purify EVs of astrocytic origin, we assessed the expression of a particular surface marker for astrocytes, the glutamate aspartate transporter (GLAST), on EVs via flow cytometry (Figure 2). The initial total EVs (TEVs) suspension was captured using the Exo-Flow beads and a biotinylated antibody targeting the GLAST on EV surfaces. Only singlets were considered during analyses. FSC vs. FITC intensity plots showed that a negligible number of particles were FITC-positive in the no-EVs control. In contrast, 100% of the particles were FITC-positive in the EVs-containing sample. The analysis showed a pure and rich ADEV subpopulation required to evaluate EV GFAP content further.

### 2.4. Western Blot Analyses of EV GFAP Levels

**Figure 3:**
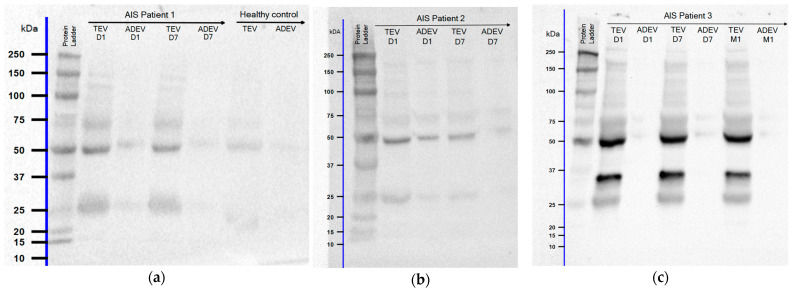
Representative blots of total-(TEV) and astrocyte-derived EV (ADEV) aliquots of three AIS patients (**a**–**c**) and one healthy control (**a**) probed with the anti-GFAP antibody are shown. Approximate molecular weight markers in kilodaltons (kDa) are labeled adjacently on the left. GFAP protein bands were observed with the indicated antibody: full-length GFAP and additional GFAP bands near 25 kDa molecular weight were detected (**a**–**c**). Bands near 37 kDa molecular weight detected in TEVs of a few patients (**c**) are also shown.

To evaluate the presence of GFAP as the EV cargo in stroke-associated samples, TEV and ADEV aliquots of AIS patients were immunoblotted and compared to healthy controls (Figure 3). When blots were probed with the anti-GFAP antibody, full-length GFAP protein at ≈50 kDa was detected in TEVs and ADEVs of all patients examined in aliquots drawn D1, D7, and M1 after injury. An additional band under the full-length GFAP, near the 37 kDa marker weight, was also visible in TEVs of 7 patients but not in ADEVs, except one aliquot at D1. We also observed bands near the 25 kDa marker weight in all TEVs and most ADEVs of patients (n = 13). These additional bands may suggest an up-regulation of a specific GFAP isoform or the presence of GFAP BDPs.

In the following, during the analysis, we focused on the full-length GFAP. The ≈50 kDa GFAP band was not detected in ADEV samples of three healthy controls; we considered a band intensity of 0 in these cases.

**Figure 4:**
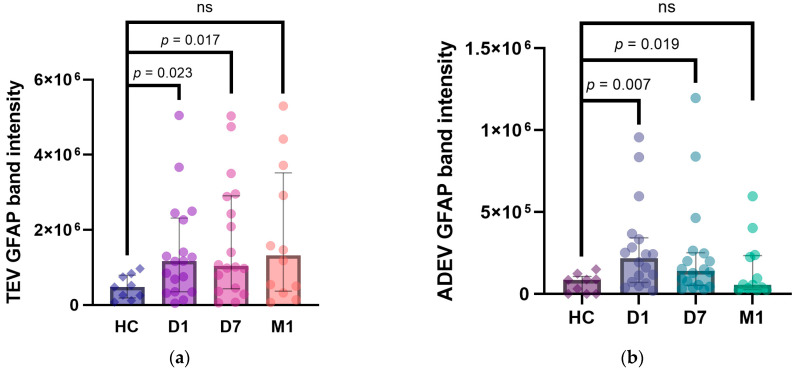
Full-length GFAP band intensities in TEVs (**a**) and ADEVs (**b**) of AIS patients and healthy controls (HC). Data are represented as individual value boxplots with median and interquartile range (IQR) (Mann–Whitney U test, ns—not significant).

To determine whether stroke affected the amount of GFAP in TEVs and ADEVs, the intensity of GFAP bands in AIS patients (D1, D7: n = 18, M1: n = 12) was compared to that of healthy control individuals (n = 9) (Figure 4). We observed a significantly higher expression of full-length GFAP-positive protein in EV aliquots of stroke patients drawn at D1 and D7 after symptoms onset compared to healthy controls but not at M1 (median, interquartile range (IQR); TEVs at D1: 1.16 × 10^6^ (0.43 × 10^6^–2.05 × 10^6^) vs. 0.47 × 10^6^ (0.25 × 10^6^–0.76 × 10^6^), *p* = 0.023; D7: 1.04 × 10^6^ (0.53 × 10^6^–2.77 × 10^6^) vs. 0.47 × 10^6^ (0.25 × 10^6^–0.76 × 10^6^), *p* = 0.017; M1: 1.32 × 10^6^ (0.45 × 10^6^–3.11 × 10^6^) vs. 0.47 × 10^6^ (0.25 × 10^6^–0.76 × 10^6^), *p* = 0.06; ADEVs at D1: 0.21 × 10^6^ (0.08 × 10^6^–0.32 × 10^6^) vs. 0.08 × 10^6^ (0–0.08 × 10^6^), *p* = 0.007; D7: 0.14 × 10^6^ (0.06 × 10^6^–0.23 × 10^6^) vs. 0.08 × 10^6^ (0–0.08 × 10^6^), *p* = 0.019; M1: 0.05 × 10^6^ (0.03 × 10^6^–0.22 × 10^6^) vs. 0.08 × 10^6^ (0–0.08 × 10^6^), *p* = 0.344).

Then, we separated the patients into those who received thrombolysis (D1, D7: n = 9, M1: n = 6) and those who did not (D1, D7: n = 9, M1: n = 6). The two groups had no significant difference regarding full-length EV GFAP (TEVs at D1, D7, and M1, respectively: *p* = 0.062, *p* = 0.136, *p* = 0.240; ADEVs at D1, D7, and M1, respectively: *p* = 0.489, *p* > 0.999, *p* = 0.588).

### 2.5. Temporal Profile of EV GFAP

**Figure 5:**
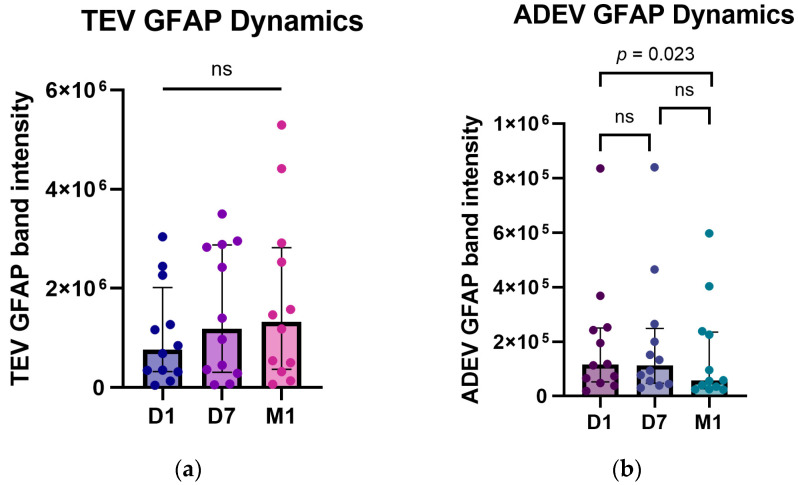
Full-length GFAP band intensities in TEVs (**a**) and ADEVs (**b**) during the patients’ follow-up: D1, D7, and M1. Data are represented as individual value boxplots with median and IQR (Friedman’s ANOVA, Dunn’s post hoc).

Next, to assess the temporal profile of post-stroke EV GFAP levels, we measured full-length GFAP band intensities in TEV and ADEV samples collected longitudinally at D1, D7, and M1 (Figure 5). The Friedman’s ANOVA test demonstrated significant differences in GFAP band intensities in ADEVs (n = 12, *p* = 0.027) but not TEVs (n = 12, *p* = 0.124) at the studied time points. Further, the post hoc Dunn’s analysis showed a significant difference between D1 and M1 (z = 2.65, *p* = 0.023) but no between D1 and D7, D7 and M1 in ADEVs.

### 2.6. Correlations between EV GFAP and Stroke Severity (NIHSS)/Outcome (mRS)

We subsequently evaluated whether full-length GFAP band intensities increase with neurological deficit, assessed by the NIHSS score reflecting the severity of symptoms, or correlate with short-term prognosis according to the mRS score at D7 and M1 after the stroke onset. A positive relationship was observed between the NIHSS at D1 and TEV GFAP at D1 (r = 0.47, *p* = 0.049) and D7 (r = 0.50, *p* = 0.031). Moreover, the mRS at D7 significantly correlated to the TEV GFAP at D1 (r = 0.48, *p* = 0.043) and ADEV GFAP at D1 (r = 0.58, *p* = 0.010) and D7 (r = 0.57, *p* = 0.013). None of the evaluated parameters at any time point showed significant correlations with the NIHSS or mRS at M1 (Table 2).

## 3. Discussion

Astrocytes are critical factors in mediating ischemic damage, with many functions regulated by cellular cross talk through EVs. A recent study revealed preferentially increased ADEV levels over the first month (from 5 to 30 days) post-IS in humans, possibly due to their trophic support on ischemia-damaged neurons; still, the cargo of these EVs has not been assessed [45]. As a highly specific marker of astrocyte activation, the GFAP is considered a promising prognostic biomarker with clinical utility [46]. We showed that ADEV GFAP may reflect dynamic changes in the first month following brain ischemia. Until now, human studies regarding the utility of ADEV cargo in various neuropsychiatric disorders have been focused on Alzheimer’s disease [47,48,49,50], frontotemporal lobar degeneration [49,50], Parkinson’s disease and Parkinson plus syndromes [51], amyotrophic lateral sclerosis (ALS) [52], multiple sclerosis [53], schizophrenia [54,55], and TBI [56]. There are some studies assessing ADEV content in the first episodes of psychosis [57], cognitive impairment in obstructive sleep apnea [58], and type 1 diabetes mellitus patients [59]. To the best of our knowledge, this is one of the first studies exploring ADEV cargo in stroke patients.

After successful isolation of the presumed ADEV subpopulation, we detected full-length GFAP at ≈50 kDa in all aliquots of TEVs and ADEVs drawn D1, D7, and M1 after ischemic injury, as well as some additional bands near 37 kDa and 25 kDa marker weights. These additional bands may suggest an up-regulation of a specific isoform or the presence of BDPs of the GFAP. The different GFAP isoforms likely have subtype-specific functions and enhance the complexity of the astrocyte cytoskeleton [13]. The GFAP-α (432 amino acids, aa) is the most commonly detected splice variant in the brain and spinal cord and is responsible for about 90% of GFAP production [60]. The other minor isoforms (β: >432 aa; γ: >321 aa, <432; δ/ε-rat/human homolog: 431 aa; κ: 438 aa; GFAP+1 (Δexon6: <347 aa; Δ164: <366 aa; Δ135: 374 aa; Δexon7: <418 aa), ζ: >432 aa, λ: 472 aa and µ: ≈21 kDa protein [22,60,61,62,63,64,65,66]) are not completely understood. GFAP is susceptible to proteolysis by caspases and calpains, which may mediate neuronal cell death both in vivo and in vitro [67]. Recently, its fragmentation patterns have been characterized in a rodent model of TBI and CSF samples from severe TBI patients. First, GFAP was fragmented at both the C- and N-terminals by calpain, resulting in GFAP BDPs ranging from 40 to 46 kDa, then 38 kDa [68]. The 38 kDa GFAP band marks the limit of calpain digestion and is a remarkably stable form of GFAP in dead or dying astrocytes [69]. When only caspases were activated but not calpain, an N-terminal BDP of 22 kDa and a C-terminal BDP of 20 kDa were generated from full-length GFAP. In CSF samples of TBI patients collected within 24 h, an increase of 38 kDa BDPs was observed [68]. A previous study also noted the calpain-mediated fragmentation of GFAP and the resulting BDPs after a TBI event [70]. Similarly, increased fragmented GFAP levels were detected in the spinal cord of patients with ALS (45 kDa, 37 kDa, and 36 kDa) [71] and in CSF of patients during the acute phase after spinal cord injury (SCI) (38–44 kDa) [72]. In Alexander disease, GFAP showed degradation products ranging between 25 and 35 kDa in both patients and mouse samples, with a 26 kDa band being the most abundant proteolytic fragment detected in four of the five cases examined [73]. An earlier study revealed two major degradation products of about 24 and 26 kDa [74]. In an Alzheimer’s disease brain, active caspase-3 cleaves GFAP at a unique DLTD266 site, generating about 30 and 20 kDa products [75]. In addition to caspase and calpain-mediated fragmentation, there is evidence for the involvement of both the autophagy and proteasome pathways in the degradation of GFAP [76,77]. These previous findings propose that GFAP BDPs are likely present in TBI, ALS, SCI, Alexander disease, and Alzheimer’s disease-associated biofluids; here, we suggest the presence of these truncated forms in the TEV population and ADEV subpopulation of IS patients. However, the mechanisms and the significance of the different degradation pathways of GFAP have yet to be determined. It is still uncertain whether these BDPs have any pathological significance in stroke. It is also possible that these fragments normally exist, and when GFAP levels are already elevated in the context of disease, their detection threshold is more easily exceeded [73]. We did not have sufficient data to analyze whether there are associations between the ≈25 kDa length GFAP and any evaluated parameters. Therefore, we cannot determine if GFAP BDPs have the potential to be possible biomarkers in IS. It should also be mentioned that while many GFAP-specific antibodies are available, few, if any, of them have defined epitopes. Commonly used monoclonal anti-GFAP antibodies recognizing specific epitopes of GFAP were characterized to verify their use in recognizing different isoforms of GFAP. In addition to the full-length protein, SMI-21 and 2.2B10 detected GFAP degradation products in samples from Alexander disease mice and humans [73].

Post-stroke TEV and ADEV aliquots drawn at D1 and D7 but not M1 showed a significant increase in full- length GFAP when compared to the control group. These results support previous findings as serum levels of GFAP are typically low in healthy individuals. When it leaks from the brain into the periphery, the resulting increased GFAP levels indicate structural damage to the brain, regardless of BBB functional status [78]. After vessel occlusion, the gradual occurrence of astrocyte damage and BBB disintegration (usually not before 6 to 12 h after stroke onset) leads to a delayed release of GFAP into the plasma, not reaching peak concentrations before 48–96 h after symptoms onset [18,79,80,81,82]. However, a recent study related an earlier peak of GFAP at 24 h [83]. Wunderlich et al. reported that serum GFAP concentrations remain increased at a lower level for at least 5 days after stroke onset [79]. This may indicate further GFAP release, probably reflecting glial scar formation [84]. In our study, the intensities of the full-length GFAP in ADEVs differed significantly during the patients’ follow-up. It has been suggested that the release kinetics of GFAP depend on the presence of a permanent brain infarction. Even in the case of critical perfusion deficits (e.g., hemispheric stroke syndromes), if cellular necrosis has not occurred yet, GFAP levels do not increase in the very early phase of IS [18,25]. Similarly, functional damage on the cellular level caused by a transient perfusion deficit or a successful recanalization of an initially occluded middle cerebral artery by intravenous (IV) thrombolysis is not associated with detectable GFAP values in the bloodstream or results in a slight increase of serum GFAP compared to patients with persistent occlusion [18,79]. We found no significant differences between patients who received thrombolysis and those who did not regarding full-length GFAP band intensities at all time points studied. One of the reasons may be that five out of the nine patients receiving thrombolysis had ischemic lesions on CT scans at D1 after stroke onset. Similarly, a recent study assessing ADEV levels in IS patients—but not their cargo—found no significant differences between participants who did or did not receive IV tPA (alteplase) [45].

Previous research has shown that there is a relationship between high GFAP concentrations in serum [35,79,81,85,86,87] or CSF [88,89], the extent of brain damage, and the severity of the neurological deficit (assessed by the NIHSS) in IS. In contrast, Kathanos et al. found no associations between baseline stroke severity and plasma GFAP levels in a cohort of 121 AIS patients [90]. In our study, full-length GFAP band intensities correlated to the NIHSS at D1 only in TEVs at D1 and D7. In fact, based on NIHSS values, GFAP has better diagnostic performance in patients with NIHSS scores above the median of 14 who have more severe stroke symptoms [18]. This may explain our results as we only included patients with NIHSS scores between 6 and 11 in this study.

As GFAP release kinetics are associated with IS patients’ neurological deficits, it may be used as an additional indicator of functional outcome one month after stroke onset [86]. Our results indicated an association between the intensities of full-length GFAP bands at D1 in TEVs, respectively, at D1 and D7 in ADEVs, and the mRS at D7 but not at M1. It was previously related that serum GFAP levels improve the ability of the NIHSS score to predict poor stroke outcomes [35]. Furthermore, GFAP concentrations in CSF (mean 8.7 h after stroke onset) [89] and serum (from 72 h on, with the highest correlation at 96 h after stroke onset) [79] correlate with IS long-term outcomes according to the mRS score at three months after the stroke onset. Similarly, an association was revealed between serum levels of GFAP and stroke patients’ independence in daily living activities over a three-month follow-up, measured through specific motor and disability scores on rehabilitation scales (trunk control test, functional ambulation classification, and functional independence measure scores) [83]. Elevated serum GFAP within 24 h predicts poor functional outcomes independently at one-year post-stroke follow-up [35]. After discharge from the hospital, serum levels of GFAP have been found to correlate with short-term outcomes according to the Barthel Index score [79,81]. Regarding ischemia-induced brain injury due to large vessel occlusions treated by endovascular embolectomy, GFAP is also a promising biomarker for predicting neurological outcomes three months after symptoms onset [87].

### Limitations of the Study

Here, we explored whether GFAP packed in EVs could have any potential significance over the first month post-stroke. However, there are some limitations of this study that need to be addressed.

Considering our aim to assess biomarkers from the cargo of EVs, immunocapture techniques were our choice for EV isolation as they offer high sensitivity and specificity in particular EV subpopulation purification. Other techniques, such as transmission electron microscopy, provide insights into the size, morphology, and integrity of EVs. Among astrocyte markers, GLAST shows the most widespread expression in astrocyte subsets in most, if not all, quiescent and reactive astrocyte subpopulations [91]. Therefore, we presume our GLAST-positive EV subpopulation to be of astrocytic origin. However, we cannot exclude the possibility that GLAST-positive EVs originate from other sources [92].

Our pilot study analyzed GFAP as cargo within EVs on a relatively small sample size using western blotting, which proved to be appropriate for detecting GFAP in EVs. However, its use to quantify EV cargo may be difficult for a large patient cohort. Further analyses and techniques need to be developed to make EV cargo determination accessible in these cases.

The present study was designed based on previously published research assessing GFAP levels in serum and CSF. We explored GFAP as a cargo within EVs but did not measure its concentration in serum or CSF.

Our patients’ follow-up was only for one month, and a longer follow-up period might have revealed different results regarding the relationships between initial biomarker levels and recovery patterns. Additionally, during this period, one patient had reinfarction, two were infected with SARS-CoV-2 one week after being discharged from the clinic, and three more could not manage to visit because they were transferred to other neurorehabilitation facilities.

## 4. Materials and Methods

### 4.1. Patients Enrolled and Study Design

We undertook a prospective, observational study where AIS patients admitted to the 1st and 2nd Neurology Clinics of the County Emergency Clinical Hospital of Targu Mures, Romania, were screened for eligibility between December 2021 and May 2023. We included adult patients of both sexes diagnosed with AIS in the territory of the middle cerebral artery less than 24 h from the onset of the symptoms, with a clinical stroke severity of 6–11 on the NIHSS, regardless of their previous treatment (antiplatelet, oral anticoagulant, statins) or if they received IV thrombolysis. We excluded patients with intracranial hemorrhage, including strokes that were initially ischemic but later underwent hemorrhagic transformation; stroke-mimic pathologies; transient ischemic attack, ischemic or hemorrhagic stroke in the last 12 months before admission; if received mechanical thrombectomy; dementia; febrile disorders, acute infection, e.g., SARS-CoV-2 infection; associated severe medical conditions, e.g., hematologic diseases, renal or liver failure, an active oncological disease with life expectancy <12 months, acute myocardial infarction.

**Figure 6:**
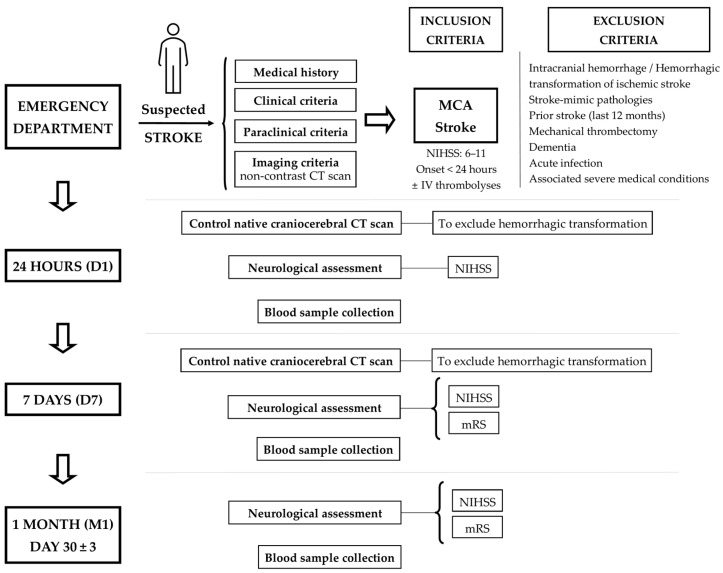
Flowchart of patient enrollment and follow-up (MCA—middle cerebral artery, CT—computer tomography).

We enrolled eighteen patients after they (or their legal representatives/family members) were informed about the study. AIS diagnosis was established by the on-call neurologist in the emergency department based on medical history and clinical, paraclinical, and imaging criteria (non-contrast craniocerebral CT scan). Time points of evaluation were 24 h (D1), 7 days (D7), and one month (M1: day 30  ±  3) after symptoms onset. A control CT scan at D1 and D7 excluded a possible hemorrhagic transformation. Clinical stroke severity was estimated at each time point using the NIHSS score, a validated scale to quantify neurological deficit following stroke [93]. The primary endpoint was the functional outcome at D7 and M1 after stroke onset, defined according to the mRS used to measure the degree of dependence in everyday life or disability in an interval from 0 (no symptoms) to 6 (dead) [94]. We dichotomized the mRS as slight (mRS 1–2) and moderate-to-severe (mRS 3–5). Data collected included demographic (age, sex) and clinical data (stroke risk factors: hypertension, diabetes mellitus, atrial fibrillation, hyperlipidemia, and smoking habit; neurological exam, NIHSS at admission and during the follow-up period; mRS at D7 and M1). The process of patient enrollment and subsequent follow-up is summarized in Figure 6. Nine healthy subjects matched for cardiovascular risk factors with demographic characteristics similar to the target group were also recruited.

The study protocol was approved by the Ethics Committee for Scientific Research of the “George Emil Palade” University of Medicine, Pharmacy, Science and Technology of Targu Mures (renewed approval no. 2303/26.04.2023), which requires all human studies to be conducted in accordance with the Declaration of Helsinki. Written informed consent was obtained from all patients (or their legal representatives/family members) before inclusion in the study.

### 4.2. Blood Collection

Peripheral venous blood samples (one vacutainer with clot activator) were obtained three times to assess the dynamic changes at 24 h, 7 days, and one month after the onset of IS. The collected samples were centrifuged in two stages within 2 h of collection to obtain plasma for the isolation of EVs: one stage at 300× *g* for 10 min (min) at 4 °C, followed by an additional centrifugation step at 2000× *g* for 20 min at 4 °C to remove cell debris. In total, 6 plasma samples in the appropriate volume were cryopreserved at −80 °C until further use.

### 4.3. Isolation and Identification of EVs from Plasma Samples

EVs were isolated using the ExoQuick^®^ ULTRA EV precipitation kit (System Biosciences, Palo Alto, CA, USA, cat. no. EQULTRA-20A-1) according to the manufacturer’s protocols [95]. First, the thawed plasma samples were centrifuged at 3000× *g* for 15 min to remove cellular debris. Then, 250 µL of supernatant was transferred to a sterile tube, mixed with 67 µL of ExoQuick reagent, and incubated for 30 min at 4 °C, followed by a 10 min centrifugation at 3000× *g* at room temperature (RT). The EVs appeared as beige or white pellets at the bottom of the tube. The supernatant was discarded, the resultant pellet was resuspended in buffer (200 µL Buffer B, followed by 200 μL Buffer A of the kit in use), and the entire content was loaded to pre-washed purification columns (with another 100 μL Buffer B added previously). The samples were mixed for 5 min on a rotating shaker at RT. Purified total EVs were collected by centrifugation at 1000× *g* for 30 s (seconds) at RT.

### 4.4. Purification of ADEV Subpopulation via Bead–Antibody–EVs–FITC Complexes and Flow Cytometry Analysis of Beads-Captured EVs

The obtained TEV suspension was further used to confirm the purification of EVs by assessing the presence of three general EV markers, the tetraspanins CD9, CD63, and CD81, on the surface of EVs, using the Basic Exo-Flow Capture kit (System Biosciences, Palo Alto, CA, USA, cat. no. CSFLOWBASICA-1) according to the manufacturer’s protocols [96]. This kit contains magnetic core beads and a hydrophilic polymer that allows the binding of streptavidin molecules to the surface that interacts with biotinylated antibodies. Pre-prepared streptavidin-conjugated magnetic Exo-Flow beads were incubated with anti-human CD9 (Miltenyi Biotec, cat. no. 130-103-954), anti-human CD63 (Miltenyi Biotec, cat. no. 130-100-169), and anti-human CD81 (Miltenyi Biotec, cat. no. 130-122-217) biotinylated capture antibodies for 2 h on ice, with gentle flicking every 30 min to mix. After incubation, the captured bead–antibody complexes were washed three times for 2 min in 500 µL Bead Wash Buffer (BWB), placing samples on the Exo-Flow Multifunctional Magnetic Stand (System Biosciences, Palo Alto, CA, USA, cat. no. EXOFLOW700A-1). Then, the complexes were suspended with 100 µL of total concentrated, isolated EV suspension together with 400 μL of BWB and subsequently incubated on a rotating shaker at 4 °C overnight to allow the efficient capture of EVs expressing the specific surface markers.

To validate the isolation procedure, following overnight incubation, the obtained bead–antibody–EVs complexes were washed two times in 500 µL BWB, then suspended in 240 µL of Exosome Stain Buffer and 10 µL of Exo-FITC Exosome FACS stain for 2 h on ice, with gentle flicking every 30 min to mix. After staining, the bead–antibody–EVs–FITC complexes were washed three times in 500 µL BWB to remove residue stains, then suspended in 300 µL BWB before the analyses on BD FACSAria™ III flow cytometer (BD Biosciences, San Jose, CA, USA). Beads without any biotinylated captured antibodies were used as negative controls. Data were processed and analyzed using BD FACSDiva™ v8.0 Software (BD Biosciences, San Jose, CA, USA).

The same protocol was applied to separate ADEVs based on the expression of a particular surface marker for astrocytes, the GLAST. The pre-prepared streptavidin-conjugated magnetic Exo-Flow beads were incubated with anti-human/mouse/rat GLAST (astrocyte cell surface antigen-1, ACSA-1) biotinylated capture antibody (Miltenyi Biotec, cat. no. 130-118-984). After the flow sort, the bead–antibody–EVs–FITC complexes were incubated with 300 µL Exosome Elution Buffer for 2 h at 25 °C on a rotating shaker to remove the Exo-FITC stain, obtaining the supernatant containing the intact eluted EVs of astrocyte origin. The ADEV suspensions were frozen and stored at −80 °C until further use.

### 4.5. Western Blot Analyses

A western blot assay was performed to detect GFAP as the EV cargo in samples obtained by the isolation procedures. Total and GLAST-positive EV aliquots were lysed with an equal volume of ice-cold RIPA Buffer (Abcam, Cambridge, UK, cat. no. ab156034) containing protease inhibitor phenylmethylsulfonyl fluoride (Abcam, cat. no. ab141032). After lysis, the protein amount was measured by a method based on the reaction of proteins with benzethonium chloride in a basic medium developed by Iwata and Nishikaze [97]. The mean protein concentration was 167.52 μg/mL (ranging from 17.86 to 508.41 μg/mL) in TEV samples and 21.52 μg/mL (ranging from 3.7 to 62.13 μg/mL) in ADEV samples. Next, the protein suspensions were mixed with an equal volume of 2× Laemmli Sample Buffer (Bio-Rad Laboratories, Hercules, CA, USA, cat. no. #1610737) and β-mercaptoethanol (Bio-Rad Laboratories, cat. no. #1610710) as a reducing reagent and heated at 95 °C for 2 min.

An input of 167.52 μg of TEV and 21.52 μg of ADEV protein lysate was loaded in wells from 10% Mini-PROTEAN^®^ TGX Stain-Free™ Protein Gels (Bio-Rad Laboratories, cat. no. #4568034, 10 well, 50 µL) for protein electrophoresis with Tris/Glycine/SDS Running Buffer (Bio-Rad Laboratories, cat. no. #1610732) in the Mini-PROTEAN^®^ Tetra Vertical Electrophoresis Cell System (Bio-Rad Laboratories, Hercules, CA, USA). Next, the separated proteins were transferred from sodium dodecyl sulfate (SDS)-polyacrylamide gels to polyvinylidene difluoride (PVDF) membranes using the Trans-Blot^®^ Turbo™ Transfer Pack (Bio-Rad Laboratories, cat. no. #1704156) with the Trans-Blot^®^ Turbo™ Transfer System (Bio-Rad Laboratories, Hercules, CA, USA). Immunodetection of EV proteins was performed with primary rabbit anti-human GFAP antibody (Bio-Rad Laboratories, cat. no. MCA6305) and goat anti-rabbit IgG (H+L) cross-adsorbed secondary antibody, HRP conjugate (Thermo Fisher Scientific, Waltham, MA, USA, cat. no. G-21234) in the presence of luminol and peroxide, using the Clarity™ Western ECL Substrate kit (Bio-Rad Laboratories, cat. no. #1705061). The chemiluminescent detection, image analysis, and densitometric quantification of band intensity were performed by the ChemiDoc XRS+ System (Bio-Rad Laboratories, Hercules, CA, USA) and ImageLab™ Software version 6.1.0 (Bio-Rad Laboratories, Hercules, CA, USA).

### 4.6. Statistical Analyses

All statistical analyses were performed using GraphPad Prism version 10.2.0 for macOS (GraphPad Software, Boston, MA, USA) and Microsoft^®^ Excel for Mac Version 16.83. Descriptive statistics for variables were reported as mean ± standard deviation (SD), median and IQR, median and min–max values, or as absolute number (n) and percentage (%). Standard differences (d) were used to compare baseline characteristics between patients and controls. The ideal d-value is <0.1; however, for small biomarker studies with n ≤ 20, an additional or missing variable between the patient and control cohorts can result in a d-value of ≥0.1 [98]. Continuous variables were compared using the non-parametric Mann–Whitney U and Friedman’s ANOVA tests. The Mann–Whitney U test was used to determine whether EV GFAP band intensities differed between stroke patients and healthy controls and between patients who received IV thrombolysis and those who did not. The Friedman’s ANOVA test was used to determine differences in EV GFAP band intensities across the follow-up period (D1, D7, and M1). The Dunn’s post hoc test was used to identify which pairs of time points differed significantly from each other (D1–D7, D7–M1, D1–M1). The Spearman’s rank correlation coefficient (r) was used to determine associations between EV GFAP band intensities and NIHSS (stroke severity)/mRS (stroke outcome) scores for each time point. A *p*-value < 0.05 was considered significant.

## 5. Conclusions

In summary, assessing EV proteins could provide a new platform that dynamically reflects the transition from brain injury to repair within the first month post-ischemia in vivo. Here, we confirmed the presence of GFAP in EVs, which could be a step forward in stroke research. Future studies await to elucidate whether EVs of different origins and their cargo protein can be viable biomarkers in stroke.

## Figures and Tables

**Table 1:** Clinical characteristics of the study population.

Variable		AIS Patients (n = 18)	Healthy Controls (n = 9)	d
Prior vascular risk factors, n (%)	Hypertension	18 (100%)	6 (66.6%)	1
	Diabetes mellitus	9 (50%)	4 (44.4%)	0.11
	Hyperlipidemia	4 (22.2%)	2 (22.2%)	0.00
	Atrial fibrillation	3 (16.6%)	1 (11.1%)	0.16
	Smoking habit	3 (16.6%)	2 (22.2%)	0.14
Pre-stroke treatment, n (%)	Antiplatelet agents	9 (50%)	N.A.	N.A.
	Anticoagulants	1 (5.5%)	N.A.	N.A.
	Statins	1 (5.5%)	N.A.	N.A.
Acute stroke treatment, n (%)	IV thrombolysis	9 (50%)	N.A.	N.A.
NIHSS, median, min–max	D1	7.5 (6–11)	N.A.	N.A.
	D7	5.5 (1–10)	N.A.	N.A.
	M1 (n = 12)	2 (1–7)	N.A.	N.A.
Etiological diagnosis (TOAST classification), n (%)	Large artery atherosclerosis	14 (77.7%)	N.A.	N.A.
	Cardioembolism	4 (22.3%)	N.A.	N.A.
	Small artery occlusion	0	N.A.	N.A.
Injured hemisphere, n (%)	Left	11 (61.1%)	N.A.	N.A.
mRS, D7, n (%)	1–2	3 (16.7%)	N.A.	N.A.
	3–5	15 (83.3%)	N.A.	N.A.
mRS, M1 (n = 12), n (%)	1–2	9 (75%)	N.A.	N.A.
	3–5	3 (25%)	N.A.	N.A.

Abbreviations: AIS—acute ischemic stroke, IV—intravenous, NIHSS—National Institutes of Health Stroke Scale, mRS—modified Rankin Scale; D1—24 h, D7—7 days, M1—one month after stroke onset; d—standardized difference.

**Table 2:** Associations between EV GFAP and stroke severity and outcome.

EV GFAP/Time Points		Scales/Time Points		r	*p*
TEV GFAP	D1	NIHSS	D1	0.47	0.049 *
			D7	0.45	0.057
			M1	0.24	0.435
		mRS	D7	0.48	0.043 *
			M1	0.30	0.355
	D7	NIHSS	D1	0.50	0.031 *
			D7	0.345	0.160
			M1	0.34	0.276
		mRS	D7	0.15	0.548
			M1	0.40	0.203
	M1	NIHSS	D1	0.40	0.189
			D7	0.15	0.619
			M1	0.42	0.164
		mRS	D7	0.15	0.638
			M1	0.37	0.235
ADEV GFAP	D1	NIHSS	D1	0.16	0.501
			D7	0.30	0.220
			M1	0.18	0.569
		mRS	D7	0.58	0.010 *
			M1	0.10	0.789
	D7	NIHSS	D1	0.07	0.779
			D7	0.212	0.398
			M1	0.29	0.350
		mRS	D7	0.57	0.013 *
			M1	0.13	0.706
	M1	NIHSS	D1	0.17	0.581
			D7	−0.19	0.535
			M1	0.06	0.841
		mRS	D7	0.27	0.389
			M1	−0.03	0.961

Spearman correlation, * significant; n = 18 (D1, D7); n = 12 (M1).

## Data Availability

Data are contained in the article and are available on request from the corresponding author.

## Abbreviations

aa	amino acids
ACSA-1	astrocyte cell surface antigen-1
ADEVs	astrocyte-derived extracellular vesicles
AIS	acute ischemic stroke
ALS	amyotrophic lateral sclerosis
BBB	blood–brain barrier
BDPs	breakdown products
BWB	Bead Wash Buffer
cat. no.	catalog number
CNS	central nervous system
CSF	cerebrospinal fluid
CT	computed tomography
d	standardized difference
e.g.	for example
EVs	extracellular vesicles
FITC	fluorescein isothiocyanate
FSC	forward scatter
GFAP	glial fibrillary acidic protein
GLAST	glutamate aspartate transporter
HC	healthy controls
IQR	interquartile range
IS	ischemic stroke
IV	intravenous
kDa	kilodaltons
MCA	middle cerebral artery
min	minutes
mRS	modified Rankin Scale
NIHSS	National Institutes of Health Stroke Scale
ns	not significant
NVU	neurovascular unit
PVDF	polyvinylidene difluoride
RT	room temperature
SCI	spinal cord injury
SD	standard deviation
SDS	sodium dodecyl sulfate
sec	seconds
TBI	traumatic brain injury
TEVs	total extracellular vesicles
vs.	versus
